# Orthodontic Fixed Retainer and Unwanted Movements of Lower Anterior Teeth: A Case Report

**DOI:** 10.1155/2022/3100360

**Published:** 2022-09-02

**Authors:** Maria Francesca Sfondrini, Maurizio Pascadopoli, Sergio Beccari, Giovanna Beccari, Cinzia Rizzi, Paola Gandini, Andrea Scribante

**Affiliations:** Unit of Orthodontics and Pediatric Dentistry, Section of Dentistry, Department of Clinical, Surgical, Diagnostic and Pediatric Sciences, University of Pavia, Pavia 27100, Italy

## Abstract

The use of fixed retainers at the end of an orthodontic treatment has become a standard practice. Nonetheless, orthodontic relapse can still occur, requiring retreatment in the most severe cases. This case report describes a patient with a mandibular canine to canine fixed retainer presenting uncontrolled torque on all lower anterior teeth, probably due to tongue thrust and/or activation of the wire. Multibracket orthodontic treatment was performed, and an orthodontic lingual sectional was used to control (reposition) the root movement of the lower right cuspid. This case highlights the need for clinicians and patients to be aware of the potential problems associated with bonded retainers. In addition, patients with an orthodontic fixed retainer need regular short-term observation by an orthodontist in order to detect any adverse movements and long-term control by a general dentist.

## 1. Introduction

Stability of orthodontic treatment is an issue of great concern among clinicians. In fact, to prevent relapse at the end of the therapy, various types of retainers are available, both fixed and removable, and should be used in every patient for a certain amount of time or permanently [1, 2]. The popular use of these devices is also justified by the potential post-treatment alterations and the increased expectancy for a perfect and esthetic permanent outcome [2].

Many orthodontists employ fixed retainers for this purpose as they seem to be the best choice to preserve long-term results. As a matter of fact, they are really effective in maintaining alignment of the anterior region without the patients' compliance [3]. Nowadays, the most common fixed retainers are those bonded to the lingual surfaces of the canines only and those bonded to all six anterior teeth [4].

It was noted that a higher percentage of patients with retainers bonded to the canines only showed incisal irregularities after a five-year follow-up, compared to those with retainers bonded to all anterior teeth. Consequently, there has been a greater use of the latter [5]. The most common is a flexible spiral wire, or similarly, dead soft wires of various sizes that can be bonded to each anterior element [2, 4].

These devices have been reported to be reliable and safe; nevertheless, they can have disadvantages and can lead to complications. For example, they can undergo fracture or detachment on single to multiple teeth [6]. A less common, but really dangerous, problem is the unwanted tooth movement, such as crown displacement or torque movements [7]. According to the recent literature, this unwanted movement could also be promoted by tongue pressure, incorrect swallowing, and other wrong habits [8]. If this kind of complication is detected earlier, it is possible to prevent damage to bone and periodontal tissue, by means of preventive measures (e.g., speech therapy, essix retainers, or other mobile retention appliances). On the other hand, if they are detected too late, they can end in biologic damage and retreatment might be requested [9].

These movements cannot be considered an orthodontic relapse, as they do not show similarity to the initial malocclusion. Such alterations are attributed to a distortion or activation of the wire, caused by a not yet known mechanism [10]. Katsaros et al. [1] were the first to describe unwanted tooth movements in the presence of a fixed mandibular retainer. Later, many similar cases have been reported. For instance, Pazera et al. [9] reported the case of a mandibular canine with increased root torque. Furthermore, Singh showed the case of a canine completely avulsed [11].

This case report presents a severe complication of a mandibular fixed retainer in which the right canine presented increased lingual root torque, while the remaining teeth bonded to the retainer showed an unwanted movement following a spiral form.

## 2. Case Presentation

This case report presents the retreatment of a woman who came in for a consultation due to the unusual position of her lower right canine. The visit was done three years from the end of a previous orthodontic treatment. The therapy lasted one year and ended with the bonding of a multibraided fixed mandibular retainer applied to all six anterior teeth. The present case report has been approved by the Unit Internal Review Board (number: 2019 0522).

### 2.1. Diagnosis and Etiology

**Figure 1:**
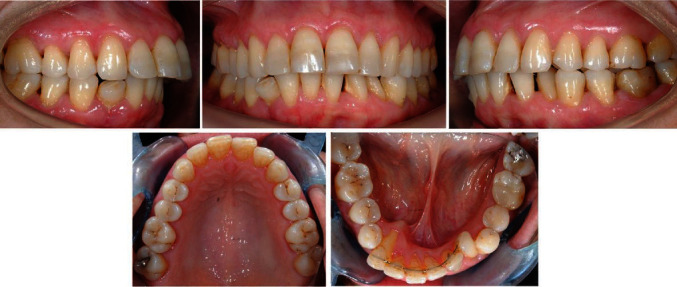
Initial intraoral photographs.

A 58-year-old patient presented molar and canine Class II relationships on both sides (Figure 1). Maxillary and mandibular interincisal midlines did not coincide. Clinical intraoral examination showed the maxillary arch quite aligned, with mild rotations of the left incisors. On the other hand, the mandibular arch presented a multibraided fixed retainer bonded to all six anterior teeth with the right canine root lingually torqued. The root was exposed, almost revealing the apex, and the element was not vital. Elements 3.2 and 3.3 resulted retroclined, withdiastemas between elements 3.1–3.2 and 3.2–3.3, a 90° rotation of 3.3. Calculus in the fifth sextant, multiple recessions, and restorations were present.

**Figure 2:**
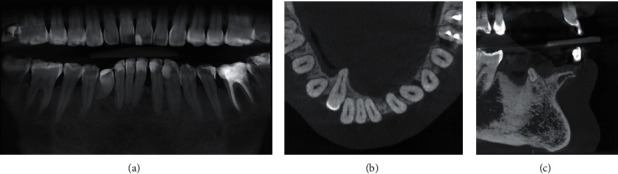
CBCT initial images: coronal (a); axial (b); and sagittal (c) sections.

A cone-beam computed tomography (Orthophos SL 3D, Sirona, Bensheim, Germany) was performed, confirming the former periodontal findings (Figure 2(a)). The root of the right canine was not covered by cortical bone anymore (Figures 2(b) and 2(c)).

### 2.2. Treatment Alternatives

Clinicians suggested different solutions to the patient. The first alternative involved the extraction of all lower incisors and the right canine, followed by the placement of dental implants with guided surgery and, finally, a prosthetic rehabilitation. However, this alternative would have not solved the rotation of element 3.3. The second alternative considered every other prosthetic solution without implant placement, but it would have led to treatment failure as extracting lower cuspids would have meant removing the anchor teeth or pillars for the prosthetic rehabilitation. These alternatives were too invasive and radical, in particular, the former alternative could have led to failures and peri-implantitis.

The most conservative solution was fixed orthodontic treatment with multibracket appliance aiming at preserving the affected teeth. This option was the least invasive, but it could have been complex from a biomechanical point of view and challenging for the retrieval of the right canine, with poor prognosis. Consequently, after being informed of all risks and having signed an informed consent, the patient chose to undergo orthodontic treatment only for the lower arch.

### 2.3. Treatment Objectives

The primary objective was to correctly reposition the mandibular right canine, in order to avoid the placement of a dental implant or fixed dental bridge. As the patient requested a noninvasive treatment, it was decided to start fixed orthodontic treatment only on the lower arch.

### 2.4. Treatment Progress

**Figure 3:**
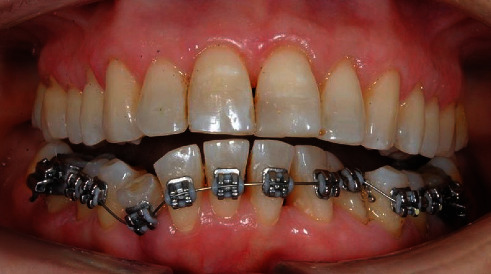
0.012-in NiTi archwire.

**Figure 4:**
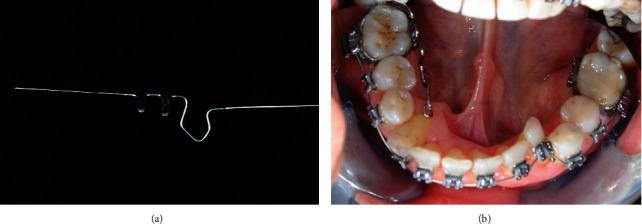
Orthodontic lingual sectional (a); lingual sectional inserted on lingual tubes (b).

**Figure 5:**
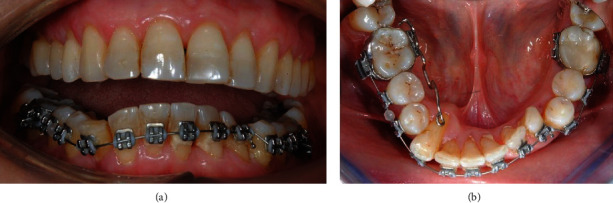
0.014-in NiTi 3M (a); orthodontic sectional (b).

After the removal of the fixed retainer, periodontal probing, supra and subgingival professional oral hygiene was performed. Periodontal probing and supragingival oral hygiene sessions were repeated if needed for calculus accumulations during all orthodontic treatment. A multibracket treatment was performed with the MTB technique. Brackets (3M Unitek, Monrovia, CA, USA) were bonded from elements 3.5 to 4.5, and molar bands with double tubes (3M Unitek) were cemented to elements 3.6 and 4.6. On the lingual side of molar bands, Wilson 3D lingual tubes (Rocky Mountain Orthodontics, Denver, USA) with vertical insertion were welded. In order to obtain additional torque on the right mandibular canine, a lower second premolar bracket (3M Unitek) was bonded. A 0.012-in NiTi archwire 3M (Figure 3) was ligated. A Wilson 3D sectional archwire (Rocky Mountain Orthodontics) was inserted on element 4.6 (Figures 4(a) and 4(b)) as an additional lingual/apical force became necessary to help the root apex return in the alveolar bone base. Its mesial extremity was placed on the most apical point of the root of the right canine, distant from lingual mucosa. The lingual sectional was removed after three months of treatment. Meanwhile, the following arch sequence was used: 0.014-in NiTi (Figure 5), 0.016-in NiTi, and a 0.019 × 0.025-in NiTi (3M Unitek). Light archwires were used for a long time in order to achieve a good alignment; considering the age of the patient, continuous and light forces were exerted to respect periodontal tissues. Subsequently, a 0.019 × 0.025-in TMA (3M Unitek) was used. Additional torque was progressively added on the right canine for the next three months. Treatment continued with the addition of radicular-vestibular torque on the IV quadrant and an elastic chain (3M Unitek) applied to close spaces.

### 2.5. Treatment Results

**Figure 6:**
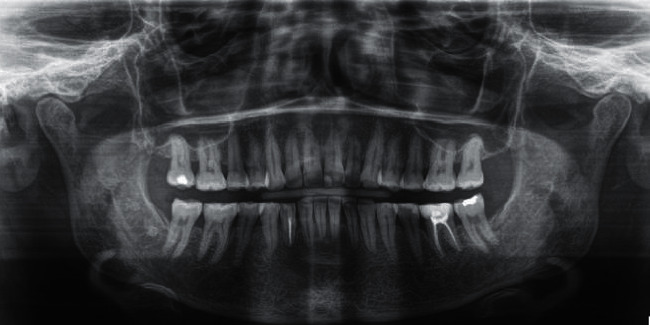
Final orthopantomography.

**Figure 7:**
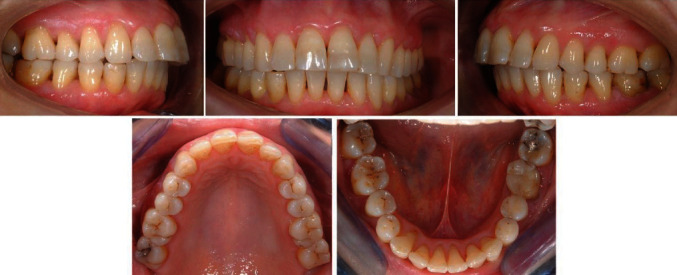
Final intraoral photographs.

The multibracket treatment lasted a year and a half. The mandibular right canine was repositioned (Figure 6) and a periodontal examination showed probing pocket depth values of 3 mm. In addition, the general dentist decided to perform root canal treatment of the canine one year after the beginning of orthodontic treatment as the tooth was not vital and not symptomatic. Elements 3.2 and 3.3 were repositioned too, and spaces were closed (Figure 7).

**Figure 8:**
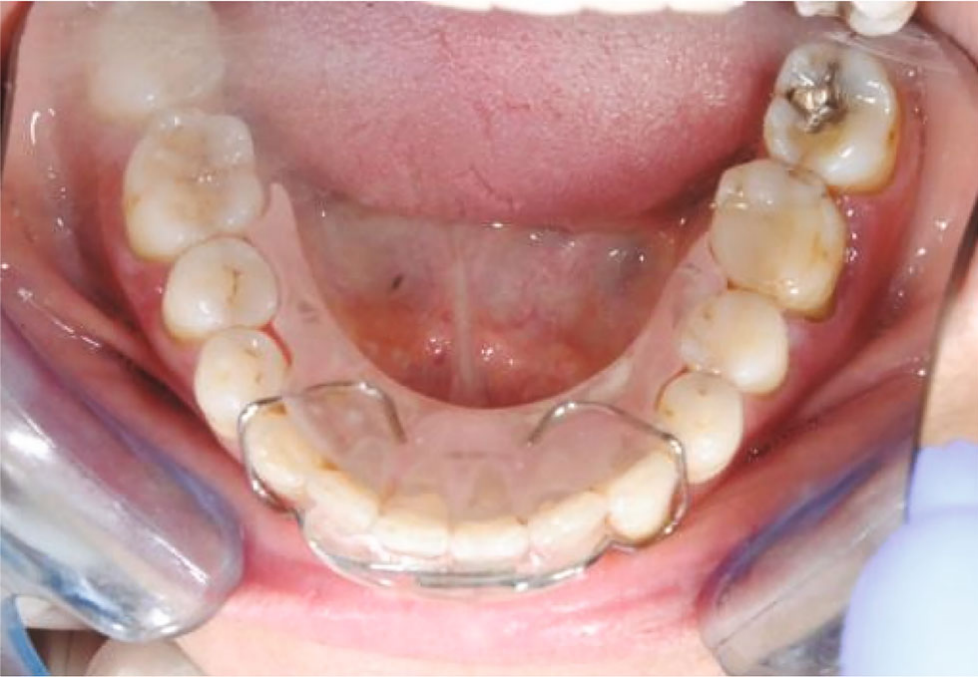
Spring retainer.

A spring retainer was delivered to the patient (Figure 8). It consisted of an anterior stainless-steel wire with vestibular and lingual resin components. The latter extended to the molars to improve stability.

**Figure 9:**
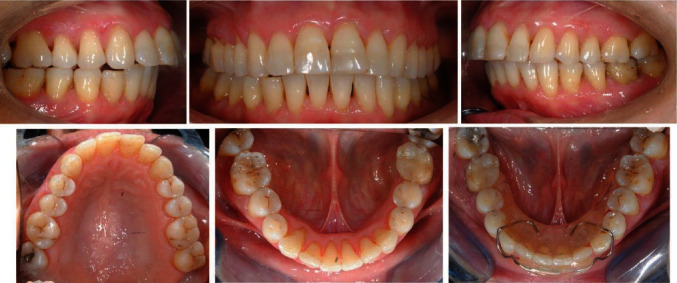
Follow-up after 18 months from the end of the orthodontic treatment.

The patient was visited after 1, 5, 12, and 18 months from the end of the orthodontic treatment. The stability of the results was observed after 18 months (Figure 9). The patient was satisfied by the result of the therapy but was aware that unwanted tooth movement could occur in the upper arch anyway as she excluded every kind of retention. The patient will continue to be under close observation through regular follow-up examinations.

## 3. Discussion

This case report illustrates the potential complications because of undesirable movements on teeth bonded with a fixed mandibular retainer. Patients with bonded flexible spiral wire retainers can incur into post-treatment changes in approximately 2.7% to 5% [1, 12]. The phenomenon could involve wire-related factors and/or functional aspects, often presenting simultaneously.

Fixed retainers are widely used at the end of active orthodontic treatment as stability cannot be predicted at the individual level [13]. Because they are compliance-free, invisible, and appear to be safe in the long term, patients generally tolerate them well [6]. Clinicians can also choose a removable device for the retention phase because they have the advantage of being easier for the patient to maintain oral hygiene. On the other hand, patient compliance is essential with removable retainers because without it, a relapse may occur. This method of retention places full responsibility on the patient in maintaining tooth alignment following orthodontic treatment [14].

Among the complications of fixed retainers, detachments, fractures, unexpected tooth movements, and difficulty in maintaining correct oral hygiene occur [6].

Many studies were conducted in order to detect the potential impact on periodontal health as bonded retainers promote plaque retention, calculus accumulation, and gingival inflammation [15]. Al-Moghrabi et al. compared fixed and removable retainers in a 4-year study, concluding that both are associated with gingival inflammation, but the former were more effective in maintaining mandibular alignment [16]. However, a recent systematic review assessed that they are compatible with periodontal health [17]. Proper oral hygiene and the correct use of dental floss underneath the retainer are crucial to avoid periodontal complications [18].

In the present case report, an uncontrolled torque on the lower frontal teeth and, in particular, on the position of the mandibular right canine was found. Such movements can be attributed to the activation or deformation of the retainer wire, which can depend on different factors, or to tongue thrust and masticatory forces.

Wire breakage can result in a loss of alignment and is more likely to occur with smaller diameter dead soft wires. Additionally, if a wire segment remains after breakage, it may lead to independent tooth movement [5]. Unexpected changes may occur despite undamaged retainers [12]. Katsaros et al. identified two types of complications: difference in torque between two adjacent lower teeth (the most common) and increased buccal inclination with the movement of a mandibular canine [1]. These post-treatment changes cannot be defined as a relapse of orthodontic treatment as they do not show similarities to the pretreatment malocclusion [3].

Clinicians usually adopt flexible spiral wire retainers on a dental cast or directly on the patients chairside in order to be passive. A mild deflection of the wire could occur during bonding procedures, leading to orthodontic forces capable of causing tooth movements. Consequently, during adapting and bonding procedures, passivity is desirable. Complications related to these factors generally arise a few months after the bonding procedure [1, 9] but can also occur later and may be the result of wire fatigue and mechanical deformation caused by masticatory forces, biting on hard food or trauma [1]. Wrong habits or incorrect use of dental floss can lead to wire deformation [5].

Sifakakis et al showed that small deflections of the wire can generate sufficiently high forces to induce unwanted tooth movement. Moreover, the composite resin layer degrades over time due to mechanical attrition, gradually exposing longer segments of the wire exposed, thus making the retainer more vulnerable to damage and failures [19, 20]. A force heavy enough to deform a retainer wire usually causes bonding failures or breakages; as a result, deformation without debonding might not be the cause of unexpected tooth movement. However, when wire deformation does not result in bond failure, teeth are likely to move [5].

In the retention phase, patients are usually supervised with dental follow-ups every 6 to 12 months for at least two years following the placement of the fixed retainer as failure rates are higher during this time period [21].

According to the literature, failure of fixed retention may result from detachments between the wire and composite resin [22], therefore adopting an appropriate bonding technique is essential [23]. Clinicians should select the best adhesive protocol and composites based on their experience and the results of the literature. Concerning the former, the enamel bond strength of universal adhesives is improved using the total etching technique [24]. In addition, a recent study demonstrated that a universal adhesive, generally employed in restorative dentistry, could be a valid alternative to the conventional orthodontic adhesive for correct adhesion of orthodontic retainers [25]. Furthermore, many studies assessed that orthodontic resin composites would be preferable than flowable composites while bonding the fixed retainer because of higher bond strength and survival rates [26–28].

Complications caused by an improper bonding procedure typically occur shortly after the fixed retainer is placed. Meanwhile, tooth movements due to mechanical deformation of the wire appears several years later [3]. As a result, patients should be informed of the potential complications during the retention phase, and regular monitoring may continue for several years. General dentists may be aware of these dental movements so that they can detect them as fast as possible [6].

In the case report of Seo et al. [29], a low position of the tongue associated with anterior thrust was likely to be responsible for undesired tooth movement, even though the patient had a fixed mandibular retainer. For this reason, besides the aforementioned wire-related factors, the role of tongue thrust should be carefully evaluated when facing undesired tooth movement and rehabilitation should be considered when facing orthodontic treatment and the retention phase [30]. However, future *in vitro* studies are desirable to assess the torsional effects of wires: this could help quantifying the contribution of mechanical force in undesired tooth movement and understanding the weight of functional aspects.

Possibly, to avoid wire-related failure factors, a removable device could be associated with the fixed retainer to prevent complications and to manage thrust tongue [5]. Currently, digital CAD/CAM workflow is available and could be considered with the intention of designing more precise retainers [31–33].

In this case, we delivered a spring retainer to the patient, as she was compliant and, in particular, she did not want a new fixed retainer in order to avoid the risk of new unwanted tooth movements. In our opinion, we preferred the spring retainer in respect to an essix retainer as the former could control the torque of the lower right canine. In addition, it had vestibular and lingual resin components at two different heights in order to prevent any movement created by tongue habits and pressure. The patient was warned to strictly attend regular visits to check stability of the results. With regard to the upper arch, the patient was aware that possible movements could occur during time, as she did not request no retreatment and no retention appliance.

Despite being a topic of great concern, a few clinical trials have been conducted to evaluate orthodontic relapse management with different appliances, and future studies should be addressed to evaluate long-term effects of fixed and mobile retainers, alone or in combination [34].

## 4. Conclusions

Orthodontists, general dentists, and patients must be aware of the potential complications of the flexible spiral retainer. Even though a retreatment is generally possible, when changes remain undetected for a long period, it could result in permanent damage. Therefore, regular and long-term monitoring of the patients is essential during the retention phase.

## Data Availability

All data are available upon request to the corresponding author.
